# Development of reconstructed intestinal micronucleus cytome (RICyt) assay in 3D human gut model for genotoxicity assessment of orally ingested substances

**DOI:** 10.1007/s00204-022-03228-y

**Published:** 2022-02-28

**Authors:** Hui Kheng Lim, Christopher Owen Hughes, Michelle Jing Sin Lim, Jia’En Jasmine Li, Moumita Rakshit, Calvin Yeo, Kern Rei Chng, Angela Li, Joanne Sheot Harn Chan, Kee Woei Ng, David Ian Leavesley, Benjamin Paul Chapman Smith

**Affiliations:** 1 Innovations in Food and Chemical Safety (IFCS) Programme, Agency for Science, Technology and Research, Singapore, Singapore; 2 Skin Research Institute of Singapore (SRIS), Agency for Science, Technology and Research, Singapore, Singapore; 3 Future Ready Food Safety Hub (a Joint Initiative of A*STAR, SFA and NTU), Nanyang Technological University, Singapore, Singapore; 4 Singapore Institute of Food and Biotechnology Innovation (SIFBI), Agency for Science, Technology and Research, Singapore, Singapore; 5 National Centre for Food Science, Singapore Food Agency, Singapore, Singapore; 6 School of Materials Science and Engineering, Nanyang Technological University, Singapore, Singapore; 7 Environmental Chemistry and Materials Centre, Nanyang Environment and Water Research Institute, Singapore, Singapore; 8 Harvard T. H. Chan School of Public Health, Harvard University, Cambridge, USA

**Keywords:** Micronuclei, Reconstructed intestine micronucleus cytome (RICyt) assay, DNA damage, Mode of cell death

## Abstract

The micronucleus (MN) assay is widely used as part of a battery of tests applied to evaluate the genotoxic potential of chemicals, including new food additives and novel food ingredients. Micronucleus assays typically utilise homogenous in vitro cell lines which poorly recapitulate the physiology, biochemistry and genomic events in the gut, the site of first contact for ingested materials. Here we have adapted and validated the MN endpoint assay protocol for use with complex 3D reconstructed intestinal microtissues; we have named this new protocol the reconstructed intestine micronucleus cytome (RICyt) assay. Our data suggest the commercial 3D microtissues replicate the physiological, biochemical and genomic responses of native human small intestine to exogenous compounds. Tissues were shown to maintain log-phase proliferation throughout the period of exposure and expressed low background MN. Analysis using the RICyt assay protocol revealed the presence of diverse cell types and nuclear anomalies (cytome) in addition to MN, indicating evidence for comprehensive DNA damage and mode(s) of cell death reported by the assay. The assay correctly identified and discriminated direct-acting clastogen, aneugen and clastogen requiring exogenous metabolic activation, and a non-genotoxic chemical. We are confident that the genotoxic response in the 3D microtissues more closely resembles the native tissues due to the inherent tissue architecture, surface area, barrier effects and tissue matrix interactions. This proof-of-concept study highlights the RICyt MN cytome assay in 3D reconstructed intestinal microtissues is a promising tool for applications in predictive toxicology.

## Introduction

Micronuclei (MN) originate from chromosomal fragments, or individualized chromosomes, that lag behind during nuclear division (Schmid 1975; Fenech 2000; Fenech and Morley 1985a, 1985b, 1986) and are commonly used to assess the genotoxic potential of chemical reagents. In the past decade, the MN assay has become increasingly popular for identifying the hazard of acquired chromosomal damage resulting from exposure to genotoxic compounds. It has become an essential step in prioritizing compounds early in the product development process, as well as meeting regulatory and statutory requirements prior to commercialisation. OECD guidelines (487 and 474) (OECD 2014, 2016b) for the in vitro and in vivo MN assay have been published, to facilitate the use of MN assays for genetic toxicity testing.

Although all MN assay protocols are based on the analysis of micronuclei frequency, they vary in terms of targeted models and technical details (Sommer et al. 2020). The key criteria for any successful micronucleus assay are (1) a low reproducible MN background and (2) sufficient cell proliferation that ensures the majority of cells analysed have undergone division. One of the most widely applied in vitro methodologies is the cytokinesis-block micronucleus (CBMN) assay which halts cytokinesis by the addition of cytochalasin B (Cyt-B). The protocol is able to discriminate between dividing and non-dividing cells, thereby improving the sensitivity of the assay by excluding cells that are not capable of presenting an MN endpoint. This measure reduces the occurrence of confounding effects resulting from variations in cell division kinetics (Fenech 2002; Kirsch-Volders and Fenech 2001). However, the CBMN assay is not suitable for certain studies, such as nanotoxicology, in which Cyt-B disrupts the cellular uptake of nanomaterial compounds (Zhang et al. 2016; Doak et al. 2012, 2009), as well as cells in post-DNA replication which escaped nuclear division (Bergoglio et al. 2013; Beau et al. 1998; Widrow et al. 1998). In vivo liver- and gastrointestinal tract-specific MN protocols have been evaluated and validated in rodents (Uno et al. 2015; Kirkland et al. 2019); tissues/animals are exposed repeatedly over a 2/4-week period to ensure that sufficient cell proliferation has occurred (Hamada et al. 2015; Martus et al. 2015). However, the use of animal studies is also facing higher scrutiny nowadays with many companies and regulators moving away from in vivo testing requirements when valid non-animal alternatives exist (Boer et al. 2020; Hardy et al. 2018; Xavier et al. 2021).

As such, focus on representative in vitro tests for “site of first contact genotoxicity” is receiving more attention. Epithelial tissues represent the body’s interface with the “outside world” and are the primary site of first contact for genotoxic materials. So far, there are limited epithelia-based genotoxicity models. The first is the buccal cytome MN assay which measures MN induced in actively dividing basal cells of oral epithelium. MN is observed in the differentiated cells of the keratinized layer at the apical buccal surface (Holland et al. 2008; Bolognesi et al. 2013). This MN assay protocol provides comprehensive information on the source of DNA damage, cytostasis and cytotoxicity (Sommer et al. 2020). The buccal MN assay is increasingly used in molecular epidemiological studies to investigate the impact of nutrition, lifestyle, and exposure to genotoxins and cytotoxins (Bolognesi and Fenech 2019; Fenech et al. 2011). This assay, however, is inappropriate for the safety and regulatory assessment of new and novel ingredients. The second, is the RHE-Skin MN test developed by Curren et al. (Dahl et al. 2011). This assay follows the CBMN approach, in which skin cell division is halted by Cyt-B and MN are scored to assess the level of genotoxicity induced by test articles. The RHE-Skin MN assay is designed for testing topically applied cosmetics or pharmaceuticals and is not suitable for food safety testing. No models currently exist for the epithelia of the gastrointestinal tract. The gut makes up a large portion of the body’s external surface (Helander and Fändriks 2014) and the small intestine is where around 90% of the digestion and adsorption of food occurs (Borgstrom et al. 1957), thus it is a major exposure site of ingested genotoxicants.

Current protocols employing in vitro 2D intestine models to screen potential toxic effects in the gut lack the critical cell–cell interactions that occur between the outer epithelial cell layer and underlying parenchyma in vivo, and also lack the extensive and intimate interactions that occur between individual cells and their pericellular environment, the extracellular matrix. It is well described that 2D cell-based models do not adequately predict gastrointestinal toxicity (Simon-Assmann et al. 2007; Noah et al. 2011; Clevers and Batlle 2013). Attempting to overcome this, others have cultivated Caco-2 (colorectal adenocarcinoma) cells on microporous Transwell™ membranes intending to provide 3D-like architecture (Hilgers et al. 1990; Hidalgo et al. 1989; Tan et al. 2018); this approach did not evolve crypt- and villus-like domains. Moreover, it is acknowledged that cancer cells (Caco-2) are not equivalent to native intestinal epithelia, rendering the value of this approach questionable. Others have used human explant tissues and laboratory animals to mimic the human small intestine. While human intestine explant cultures offer advantages over 2D cell cultures, the scarcity of normal donor human tissue and their short survival ex vivo are difficult to reconcile with routine experimental demands and requirement for robust reproducibility. Alternative animal models also have fundamental problems: they are expensive, have limited throughput, are often ethically challenging, and may not be representative of human physiology due to species differences. We propose that 3D reconstructed human epithelial (RHE) models of the intestine offer a more representative in vitro model system possessing authentic structural, biochemical, physiological, and mechanistic properties that are not available in more conventional 2D cell models of the gut. We suggest that 3D organotypic intestinal microtissues, for example EpiIntestinal™ (MatTek Corporation^®^), offer greater structural and physiological relevance, and authenticity for toxicological assessment and mechanistic research.

The 3D EpiIntestinal™ microtissues are constructed in Transwell™ devices from primary tissue obtained from consenting human donors (in accordance with MatTek Corporation IRB approval). Each microtissue construct incorporates enterocytes, Paneth cells, M cells, tuft cells, and intestinal epithelia, propagated on a basal lamina propria formed from intestinal fibroblast cells. Cultivation in vitro results in spontaneous polarized organization and differentiation, including the development of apical villus- and crypt-like domains. More than recapitulating the 3D architecture of intestinal tissue, the construct also recapitulates the biochemistry, physiology and functionality of native human small intestine in vivo (Ayehunie et al. 2018). While it has been commonly used as an in vitro model for studies of drug absorption, metabolism and inflammation in the small intestine (Ayehunie et al. 2018; Peters et al. 2019; Vaidya et al. 2019), recently the in vitro 3D-RHE intestine model has also been used to evaluate the cytotoxicity, genotoxicity and immunotoxicity of substances such as copper oxide nanoparticles (Henson et al. 2019), nanosilver (Dvorakova et al. 2015; Pinďáková et al. 2017), and food-contact materials (Bossuyt et al. 2019; Kejlova et al. 2015). The reconstructed human intestine model has been shown to exhibit physiologically-relevant tissue responses to xenobiotic compounds (Kejlová et al. 2019). Taken together, these data support a growing interest in the use of 3D-RHE microtissue constructs for food safety assessment.

Henceforth, we aimed to evaluate whether the MatTek 3D EpiIntestinal™ microtissue is a suitable technology platform to assess genotoxicity in the human gut. We have characterized EpiIntestinal™ microtissues and found the reconstructed human 3D EpiIntestinal™ technology platform recapitulates many characteristics of native human small intestine. To mimic in vivo exposure, we subjected EpiIntestinal™ microtissues to prolonged exposure to reference and test compounds, using a modified MN cytome assay protocol (Ayehunie et al. 2018; Peters et al. 2019). We optimized the protocol to support the detection and analyses of chromosomal damage. Referenced scoring criteria were adopted from published protocols (Bolognesi et al. 2013, 2015, 2017; Thomas et al. 2011, 2009). We have named our new protocol the “Reconstructed Intestine Micronucleus Cytome (RICyt) assay”.

## Materials and methods

### 3D Tissue culture

EpiIntestinal™ tissue (SMI-200-FT v2.0) was purchased from MatTek Corporation® (Ashland, MA). The EpiIntestinal™ construct is a differentiated 3D tissue model that consists of intestinal villi and crypts topology resembling the normal human small intestine. The tissue is constructed by incubating fibroblasts at 37 °C for 4–6 h in cell culture inserts (surface area = 0.6 cm^2^, membrane pore size = 0.4 μm) and primary human small intestinal epithelial cells, derived from consented donors, were overlaid and cultured in SMI-100-MM media (MatTek Corporation^®^) for 4 days submerged; and then for an additional 10 days at the air–liquid interface at 37 °C, 5% CO_2_/95% air and 98% relative humidity. The mature tissues are shipped sealed in 24-well agar-plugged plates to the designated laboratory (in this instance, Singapore). Upon receipt, the tissues are revived overnight in fresh SMI-100-MM before use in assays.

### Cell culture

TK6 cells (CRL-8015™, purchased American Type Culture Collection, ATCC, Manassas, VA) were cultured in RPMI-1640 medium supplemented with 2 mM L-Glutamine, 1 mM Sodium pyruvate, 4.5 g/L Glucose, 10 mM HEPES, 1.5 g/L NaHCO_3_ (PAN™ Biotech, Aidenbach, Germany), 10% horse serum (Life Technologies; Thermo Fisher Scientific, Waltham, MA) and incubated at 37 °C, 5% CO_2_/95% air and 98% relative humidity. Cells were passaged until sufficient numbers allowed for experimental purposes, counted, and seeded (2 × 10^5^ cells/well) into 12-well plates (Falcon^®^, Corning, Tewksbury, MA, 3.8 cm^2^/well). For the non-genotoxin vs genotoxin assay, TK6 cells were seeded (2 × 10^5^ cells/well) in 24-well plates (CELLSTAR^®^, Grenier Bio-One, 1.9 cm^2^/well). The cells were subsequently fixed in Carnoy’s solution or processed via cytocentrifugation (Thermo Fisher Scientific, Waltham, MA). One thousand cells had been scored per treated sample for MN induction. The TK6 MN data were generated from independent duplicates. Each experiment consisted of a single sample per dose.

### Test compound preparation and treatment

Reference genotoxins (mitomycin C, M7949, vinblastine sulphate, V1377, and benzo(a)pyrene, B1760) were purchased from Sigma Aldrich (St Louis, MO). Phenformin HCl (PHR1573, Supelco), purchased from Sigma Aldrich (St Louis, MO) was used as non-genotoxic negative control. Test compound stock solutions were prepared in DMSO (D161802, Sigma Aldrich, St Louis, MO). For the 3D tissues, dilutions were made in complete culture media yielding ≤ 0.1% DMSO final concentration. Media containing 0.1% DMSO (i.e. without test article) were applied to tissues to serve as the vehicle control. All 3D tissues were treated apically every alternate day (from day 0–6) and daily (from day 7–9), while basolateral wells contain untreated culture media to support the tissue growth. TK6 cells were exposed for 24 h with the test materials prior to harvest. S9 liver fraction (11-402L, MolTox, Boone, NC) was added to TK6 cells 3 h prior to benzo(a)pyrene exposure to activate cellular metabolism (Cox et al. 2016).

### Tissue dissociation

3D tissues were dissociated using human tumour dissociation kit (Miltenyl Biotec, Bergisch Gladbach, Germany), as per manufacturers’ instruction, with minor modifications. Individual 3D tissues cultured on microporous membranes were excised from the culture inserts and incubated for 15 min in cell recovery solution (Corning, Tewksbury, MA). The tissues were subsequently peeled off from the membrane with fine point forceps under stereo microscope (Leica Microsystems, Wetzlar, Germany). The peeled tissues were placed into gentleMACS C-tube (Miltenyi Biotech, Bergisch Gladbach, Germany) containing the enzyme mix described in the kit’s protocol. The tubes were then transferred into a gentleMACS octo dissociator with heater (Miltenyi Biotech, Bergisch Gladbach, Germany), and subjected to one cycle of ‘37C_m_LPDK_1’ program. Upon completion, the cell suspension was centrifuged at 1000 rpm for 5 min; the supernatant was removed and the pellet was resuspended in 2 mL of Hanks Balanced Salts Solution (HBSS). Total cell number was estimated through trypan blue dye exclusion method (Strober 2015) to determine the cell viability. The proliferation of the 3D tissue was estimated through calculation of population doubling based on the dissociated cell number at the day 1, 6 and 11 after tissue revival. The cells were then (1) fixed for micronuclei scoring or (2) processed for subsequent experimentation.

### Cytokinesis-blocked micronucleus analysis (CBMN)

The CBMN assay was conducted according to a previously published procedure (Fenech and Morley 1985b). Briefly, cytochalasin B (5474, Tocris Bioscience, Bristol, UK, 6 μg/ml) was applied to the 3D EpiIntestinal™ in vitro tissues every other day, to the apical and to the basolateral compartments. Tissues were dissociated into single cell suspension (described above), processed with mild hypotonic cold 75 mM KCl, followed by 3 fixation steps in Carnoy’s fixative (methanol/acetic acid, 16:1), supplemented with formaldehyde at 1st fixation. The cell suspension was stored in fixative at 4 °C overnight, stained with acridine orange (1:100 dilution in 1 × PBS, A1301, Invitrogen; Thermo Fisher Scientific, Waltham, MA) and scored under fluorescence microscopy (Zeiss Axioimager, equipped with triple band excitation fluorescence filter set: DAPI, FITC and Texas red). Scoring was performed following the criteria developed by Fenech et al. (Fenech et al. 2003, 2007). One thousand total cells per microtissue were scored to determine the percentage of cells with one (mononucleated) or two nuclei (binucleated). The results were generated from three independent experiments. Each experiment consisted of a single microtissue per dose.

### Micronucleus cytome assay

Tissues were exposed to test substances, harvested and dissociated into single cell suspension as described above. Cell suspension samples were divided equally; half of the cell suspension sample was fixed in Carnoy’s solution with 3 fixation steps in methanol/acetic acid 16:1, supplemented with formaldehyde at 1st fixation. The cell suspension was aged at least overnight at 4 °C to improve the quality of fixed cells. The remaining cells were incubated with 4% formaldehyde for 15 min, rinsed twice with 1 × PBS and stored in 4 °C. The Carnoy-fixed cells were stained with acridine orange immediately scored per criteria originally described by Tolbert et al. (Tolbert et al. 1992) and Thomas et al. (Thomas et al. 2009). Formaldehyde-fixed cells were stained with either DAPI alone (test compound treated) or DAPI and active caspases-3 antibody (9661S, Cell Signalling Technology, Danvers, MA) (DMSO treated). Fluorescence microscopy was used to capture images of untreated/ treated sample; high-resolution images were analysed using ImageJ software (Rasband, W.S., ImageJ, U. S. National Institutes of Health, Bethesda, Maryland, USA, https://imagej.nih.gov/ij/, 1997–2018). One thousand total cells were scored per microtissue to determine the frequency of MN and cytome. The percentage of MN and cytome at each concentration of test chemical was compared with the solvent control. The results were generated from three independent experiments. Each experiment consisted of a single microtissue per dose.

### Tissue barrier integrity assessment

#### Transepithelial electrical resistance (TEER) measurement

Changes in barrier integrity of the EpiIntestinal™ tissues were assayed using Transepithelial electrical resistance (TEER) with an EVOM volt-ohmmeter equipped with an EndOhm electrode chamber (World Precision Instruments, Sarasota, FL), according to the manufacturer’s published protocols. Raw resistance values (Ω) were converted to TEER readings (Ω cm^2^) by multiplying the raw measurements of each tissue by the surface area of the cell culture inserts (0.6 cm^2^).

#### FITC-dextran tissue permeability assay

Changes induced to the barrier function of the EpiIntestinal™ tissues were monitored using fluorescein isothiocyanate (FITC)-dextran 20 kDa (FD20, Sigma Aldrich, St Louis, MO) tissue permeability assay. The tissue intactness was measured in EpiIntestinal™ tissues after adding 3 mg/ml of FD20 to the apical culture media and incubating at 37 °C, for 2 h in 5% CO_2_/95% air. The permeation of FD20 into the basolateral culture medium was determined with a microplate reader (SpectraMax M5, Molecular Devices, San José, CA) at 485/530 nm. FD20 permeation from the donor medium to the receiver medium was normalized to FD20 that permeated through blank inserts. Tissue barrier integrity was considered intact if FD20 permeability was less than 4%.

### Histology and immunofluorescence

3D EpiIntestinal™ tissues were immersed in 15% and 30% sucrose consecutively for 1 h at room temperature, and snap-frozen in Optimal Cutting Temperature freezing medium (Tissue-Tek^®^, Torrance, CA). The samples were further processed into 25 μm-thick sections and incubated with primary antibodies against villin (sc-58897, Santa Cruz Biotechnology, Inc, Santa Cruz, CA), followed by appropriate Alexa Fluor 488- or 594-conjugated secondary antibodies (Molecular Probes; Thermo Fisher Scientific, Waltham, MA). Samples were counterstained with 4′,6-diamidino-2-phenylindole (DAPI, Sigma-Aldrich, St Louis, MO). To visualize F-actin filaments, samples were incubated with Alexa Fluor 488-labeled phalloidin (A12379; Life Technologies; Thermo Fisher Scientific, Waltham, MA) and counterstained with DAPI. Images were captured at room temperature using a fluorescence microscope (Zeiss AxioImager Z1; Carl Zeiss, Oberkochen, Germany), or confocal microscope (Olympus FV1000, Tokyo, Japan) with a Texas red, GFP, or DAPI filter. Image analysis was performed using Zeiss image analysis software, ZEN Pro Version 2.0 (Zeiss Microscopy, Jena, Germany).

### Tissue viability

Viability was assessed in EpiIntestinal™ tissues using Alamar blue (resazurin sodium salt, R7017, Sigma Aldrich, St Louis, MO). The tissues were rinsed twice with HBSS with calcium and magnesium and transferred to a 24-well plate. Alamar blue at desired working concentration was added to apical compartment of the tissues and incubated in a 37˚C, 5% CO_2_/95% air, 98% humidity, incubator for 1.5 h. The changes in fluorescence of the Alamar blue dye conversion was measured (excitation 560 nm and emission 590 nm) in each sample well using a microplate reader (SpectraMax M5, Molecular Devices, San José, CA).

### SEM

To visualize the columnar morphology of the intestinal 3D-RHE tissue, scanning electron microscopy was performed. Briefly, the tissue was fixed with a mixture of 4% paraformaldehyde (Sigma-Aldrich, St Louis, MO) and 2% glutaraldehyde (Invitrogen; Thermo Fisher Scientific, Waltham, MA) solution overnight at room temperature (RT). Subsequently, the tissue was cut into equal pieces and post-fixed/stained with 2% osmium tetraoxide (Sigma-Aldrich, St Louis, MO) for 1 h. Excess stain was removed by washing with PBS. Tissue samples were then serially dehydrated with an ascending series of ethanol (25%, 50%, 75%, 95%, 100%) 15 min each at RT. The dehydrated tissues were then immersed in hexamethyldisilazane (HMDS; Sigma Aldrich, St Louis, MO) for 10 min, followed by air drying in a desiccator. The dried samples were then mounted on to a SEM sample stub, sputtered with gold and viewed in the SEM (Jeol JSM-6360LV, Tokyo, Japan).

### Statistical analysis

All data are presented as means ± standard error of mean. Two-tailed Student’s *t* test was used to determine the significance between two groups and data was deemed significant if *p* < 0.05. All experiments were performed three times independently. Each experiment consisted of a single microtissue per dose unless otherwise indicated in figure legends.

## Results

### Characterization of 3D reconstructed human small intestine model

**Fig. 1:**
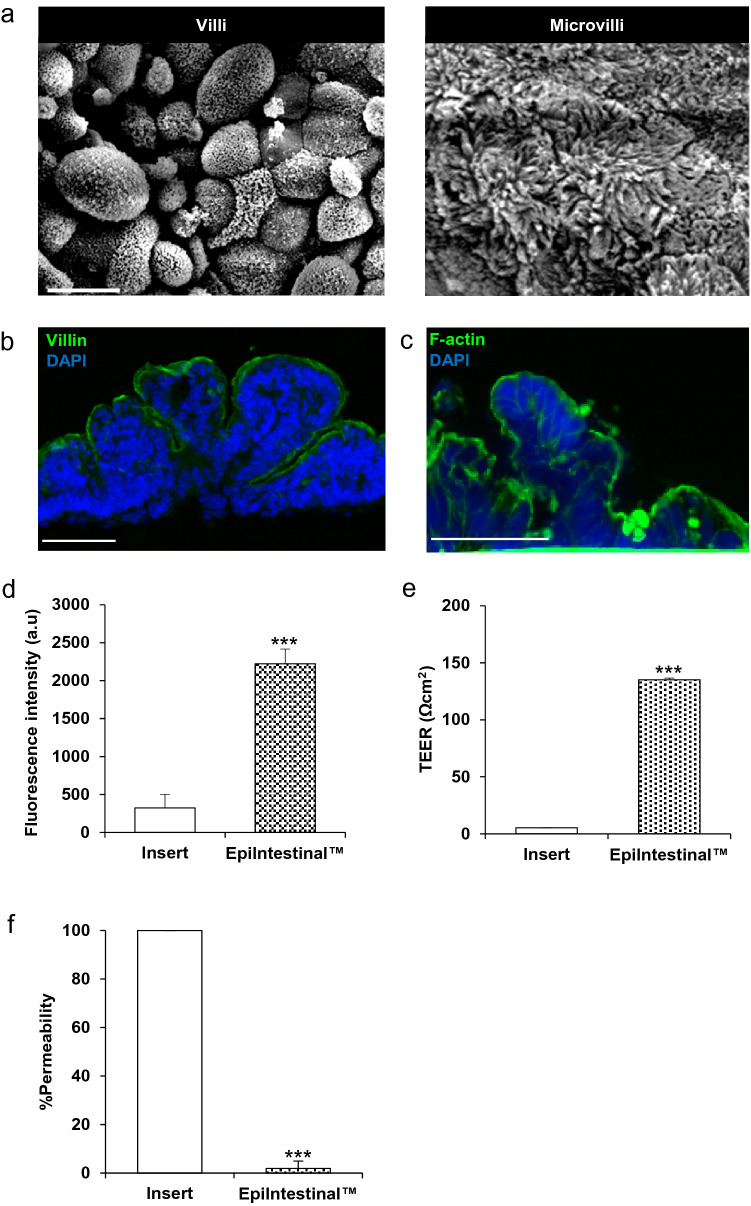
EpiIntestinal™ tissue recapitulates unique 3D tissue architecture and barrier function of normal human small intestines. **a** SEM was used to visualize villi (left panel) and microvilli (right panel) structures on EpiIntestinal™ tissue. Scale bar = 10 μm. **b** Immunofluorescence staining identifies villin (green) and DNA stain (blue) in the 3D EpiIntestinal reconstructed microtissue. Scale bar = 10 μm. **c** F-actin (green) and DAPI (blue) immunohistochemical staining of cryosections (25 µm) of OCT embedded, EpiIntestinal™ tissue. Scale bar = 20 µm. **d** Alamar blue viability assay of 3D tissue. **e** TEER measurement of tissue barrier integrity and **f** FITC-dextran assay of tissue permeability. The results from three independent experiments are shown. Each experiment consisted of a single microtissue per test condition. ***p < 0.001

The EpiIntestinal™ microtissue is a 3D-RHE technology platform designed to provide a route-appropriate and species-specific system for laboratory-based applications including biochemical, pharmacological and toxicological analyses. To assess the feasibility of utilizing EpiIntestinal™ microtissues to evaluate responses to ingested particles, compounds and genotoxic reagents via an established MN assay protocol, we first examined the structure and architecture of the reconstructed tissue using scanning electron microscopy (SEM) (Fig. 1). We confirmed the tissue was stratified with apical-basal polarization, villi structures (Fig. 1a, left panel), brush-border morphology and microvilli (Fig. 1a, right panel). Epifluorescence microscopy revealed in vivo-like tissue architecture with intestinal villi structures and columnar epithelium expressing villin (intestinal cell differentiation marker) (Fig. 1b). The presence of villi in the 3D-RHE microtissue construct provides substantially greater surface area to facilitate the uptake of test substances than is available in 2D cell culture models. Qualitative immunohistology analyses verify EpiIntestinal™ tissues exhibit apical–basal polarity with a highly developed, F-actin-rich, apical brush border of absorptive epithelial cells (Fig. 1c). The tissue constructs remain viable (Fig. 1d) and maintain barrier integrity yielding average measure of TransEpithelial Electrical Resistance (TEER) of 135 ± 1.5 Ω cm^2^ (Fig. 1e), equivalent to in vivo physiological TEER measures of 50–100 Ω cm^2^ (Amidon et al. 2000; Srinivasan et al. 2015; Takenaka et al. 2016). The TEER measured in EpiIntestinal™ tissues is significantly lower than TEER reported for Caco-2 monolayer cells (250 − 4000 Ω cm^2^) (Amidon et al. 2000). We also conducted fluorescein isothiocyanate (FITC)-dextran permeability assays, a complementary measure of barrier integrity, yielding average permeability 2.25% ± 0.8 (Fig. 1f).

### Development of MN cytome assay protocol in 3D reconstructed EpiIntestinal™ tissue

**Fig. 2:**
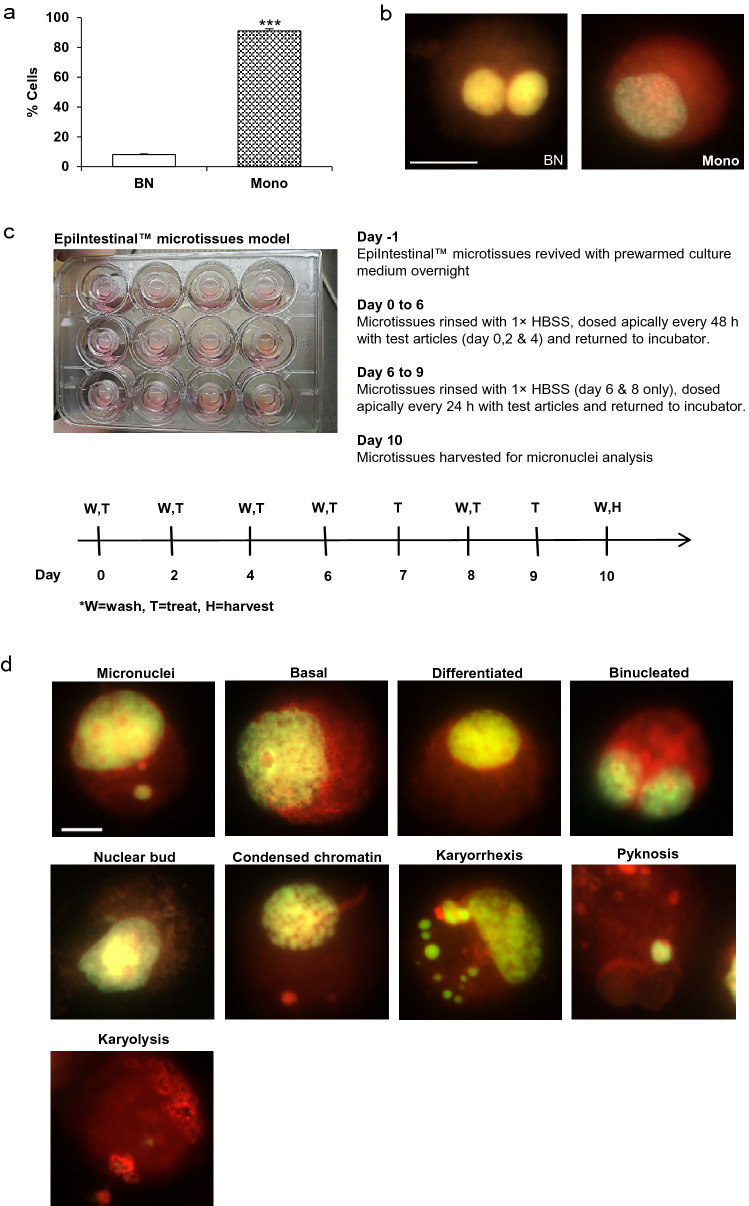
Development of MN cytome assay in EpiIntestinal™ tissue. **a** Prevalence of binucleated (BN) and mononucleated (Mono) cells dissociated from EpiIntestinal™ tissue following 10 days’ cytochalasin B exposure. One thousand total cells per microtissue were scored to determine the percentage of mononucleated and binucleated cells. **b** Microscopic images of BN and Mono cells dyed with acridine orange. Scale bar = 10 µm. **c** Treatment regimen of 3D EpiIntestinal™ tissue over 10 days. **d** Various cell types and nuclear anomalies identified and evaluated in MN cytome assay. The Mitomycin C (2 μg/ml) treated EpiIntestinal™ tissue were dissociated, fixed and differentially stained with acridine orange (see “Materials and methods”). Scale bar = 10 μm. The results from three independent experiments are shown. Each experiment consisted of a single microtissue per test condition. ***p < 0.001

OECD guidelines for the testing of chemicals and substances which are potentially genotoxic and carcinogenic, that induce genetic damage, recommend the micronucleus assay protocol (OECD 2016b). There are several versions of the test (OECD 487) designed to detect micronuclei originating from acentric chromosome fragments (i.e. lacking a centromere) in the cytoplasm of interphase cells, or whole chromosomes that are unable to migrate to the poles during the anaphase stage of cell division. The guideline allows protocols to be conducted either in the presence, or in the absence of cytokinesis blockage, depending on the intention of the treatment, types of tissue/cell models, genotoxic mechanisms, as well as whether the cell population analysed has undergone sufficient division. The in vitro cytokinesis block micronucleus (CBMN) assay is the version most commonly used (Llewellyn et al. 2020; Fenech et al. 1991; Fenech 2007). In the CBMN assay, affected cells (after cell division) are recognized by their binucleated (BN) phenotype after cell division is blocked with Cyt-B, a highly specific disruptor of microfilament assembly required for the completion of cell division (Fenech 2000; Fenech and Morley 1985a, 1985b, 1986; Carter 1967). To determine sensitivity of proliferating cells in EpiIntestinal™ microtissues to Cyt-B, we treated the 3D tissues apically and basolaterally for 10 days with 6 μg/ml of Cyt-B. Approximately eight percent (8.1%) of cells isolated from EpiIntestinal™ microtissues exposed to Cyt-B contained BN, the majority (91.9%) were phenotypically normal: i.e. mononuclear (Fig. 2a, b). Notably, the BN population is an underestimation of cell proliferation in the 3D tissue due to rapid intestinal cell turnover and shedding via the apical (luminal) surface. To provide a more comprehensive analysis of genotoxicity, mitotic dysfunction and cell death, we developed and optimised a MN cytome protocol in which the MN frequency in the mononucleated cell populations (without Cyt-B), reflects 3D EpiIntestinal™ responses to long-term treatment regimen (Fig. 2c).

In contrast to commonly used in vitro models such as TK6, or human lymphocytes, in vitro proliferation of 3D EpiIntestinal™ is not dissimilar to intestinal epithelia in vivo (Altay et al. 2019). To allow longer exposure period, our protocol uses a repetitive, 10-day test article exposure, every 48 h from day 0–6. To control dehydration and media exhaustion subsequent to sustained metabolic activity, we increased treatment frequency to 24-hourly from day 6 to day 9; tissues were harvested at day 10. Prolonging tissue exposure to test articles provides sufficient time for cells in EpiIntestinal™ tissue to traverse more than one cell cycle. Test articles were added to the apical side of the Transwell™ to recapitulate native interactions with test compounds and materials as they occur in vivo. Distinct cell phenotypes and nuclear anomalies are evident in the 3D EpiIntestinal™ MN cytome assay (Fig. 2d). These were identified using detailed criteria as described by Bolognesi, et al. (Bolognesi et al. 2013) and included micronuclei and nuclear buds, mononucleated (basal and differentiated) cells, binucleated cells, condensed chromatin, karyorrhectic, pyknotic and karyolytic cells. Our scoring protocol was adopted and modified from the approach developed by Thomas et al. (Thomas et al. 2009). One thousand total cells were scored per microtissue. The results were recorded from three independent experiments. Each experiment consisted of a single microtissue per dose.

**Fig. 3:**
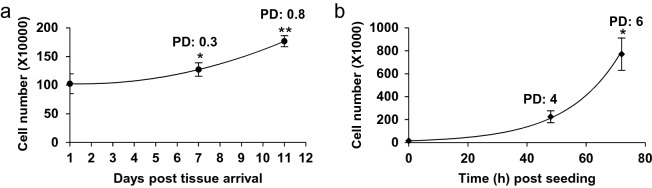
Assessment of cell proliferation in EpiIntestinal™ tissue. **a** Cell proliferation of EpiIntestinal™ tissue harvested at day 1, 7 and 11 post tissue arrival. PD = population doubling. **b** TK6 cell proliferation at 48 h and 72 h post cell seeding. The results from three independent experiments are shown. Each experiment consisted of a single microtissue per test condition. *p < 0.05 and **p < 0.01

Scorable MN are formed during anaphase of mitosis. It is necessary to demonstrate that cells scored in the assay have completed mitosis and undergone division during, or following, exposure to the test articles, to minimise false negative responses. The completion of mitosis is determined through assessment of EpiIntestinal™ tissue proliferation. We cultured the EpiIntestinal™ tissue over 11 days’ after receipt and monitored tissue growth. The EpiIntestinal™ cell number was observed to increase by 30% (population doubling, PD: 0.3) after 7 days and by 80% (PD: 0.8) after 11 days’ cultivation (Fig. 3a). In contrast, TK6 cell populations were observed to increase 400% (PD: 4) after 2 days, and 600% (PD: 6) after 3 days (Fig. 3b). It is important to note that these quantitative measures of 3D EpiIntestinal™ tissue proliferation do not account for cell shedding from the apical (luminal) intestinal epithelial surface, a manifestation of intestinal homeostasis.

### Evaluation of background micronuclei frequency in 3D EpiIntestinal™ tissue

**Fig. 4:**
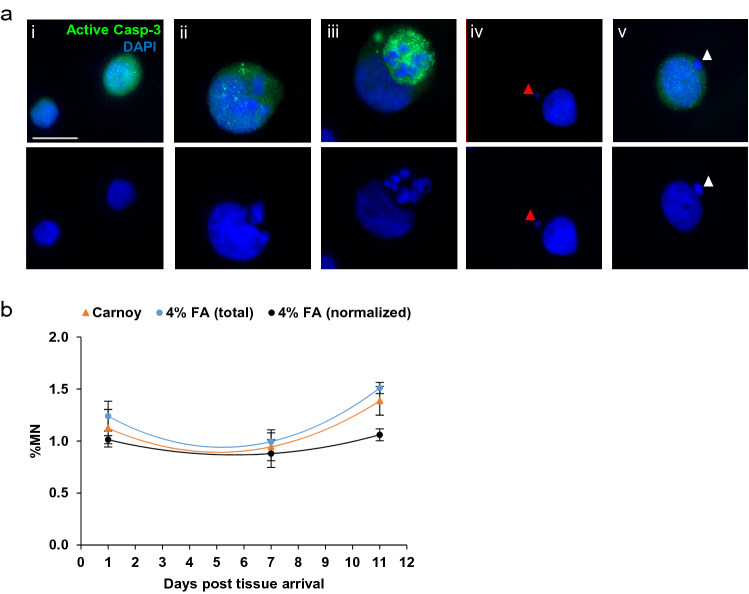
Prevalence of background micronuclei (MN) in EpiIntestinal™ tissue. **a** Immunofluorescence staining of active caspase-3 (green) and DAPI (blue) in 0.1% DMSO (solvent) treated EpiIntestinal™ tissue, differentiate early apoptotic cells (**i**–**iii**) and genuine MN (red arrow, **iv**) from DNA fragment(s) stemming from early apoptotic cells (white arrow, **v**). Scale bar = 10 µm. **b** Prevalence of basal MN in EpiIntestinal™ tissue on day 1, 7 and 11. Dissociated single cells were fixed in Carnoy’s fixative and 4% formaldehyde (FA). 4% FA (total) represents total basal MN scored from the fixed cells, while 4% FA (normalized) represents the population of MN after exclusion of DNA fragment(s) stemming from early apoptotic cells in the same samples. One thousand total cells were scored per microtissue. The results from three independent experiments are shown. Each experiment consisted of a single microtissue per test condition

A key determinant of MN assay sensitivity is the basal, or endogenous prevalence of MN in a cell culture, or a tissue model/construct. Under normal circumstances the formation of MN is a rare event, tissue homeostasis and genome stability is rigorously maintained by redundant DNA repair machinery and active cell cycle checkpoints; DNA damage is promptly repaired to ensure the quality and integrity of the genome (Jayakumar and Kasturi 2020; Luzhna et al. 2013*).* As an epithelial in vitro model system EpiIntestinal™ tissue maintains a relatively high rate of cellular proliferation, baso-apical migration, apoptosis and cell shedding, in common with native human small intestine (Bullen et al. 2006). Shed cells are not removed from the system, as they would be in native tissue by peristalsis. Thus fragments from shed cells, including DNA particles, microvesicles and apoptosomes, are sources of contamination that may be mistaken for MN, and inflate measures of background MN (Lukamowicz et al. 2011). To address this potential confounder, we optimized the exposure of EpiIntestinal™ tissue to test articles, washing EpiIntestinal™ tissue constructs with HBSS every 2 days to minimise basal MN and microvesicle contamination (Fig. 2c). We assayed for active caspase-3, to detect pre-apoptotic cells (Belloc et al. 2000; Zhang et al. 2013); the expression of active caspase-3 was detected prior to the onset of apoptosis-induced DNA fragmentation (Fig. 4a(i)) and the formation of MN. Expression intensified with increasing severity of DNA fragmentation in apoptotic cells (Fig. 4a(ii)-4a(iii)). Thus, detecting active caspase-3 is able to resolve “DNA fragment(s)” stemming from apoptotic cells (Fig. 4a(v)) from authentic MN from unrepaired DNA damages (Fig. 4a(vi)), and eliminate false positives from MN assay measures. We determined the background prevalence of MN in EpiIntestinal™ tissue after 1 day and 6 days’ exposure to 0.1% DMSO, finding the prevalence of MN was ~ 1.1%. The background prevalence of MN increased to 1.5% in tissues harvested at day 11, however, this was not significantly different to tissues exposed for shorter periods (Fig. 4b). Interestingly, when we excluded DNA fragment(s) stemming from apoptotic cells from the total MN count, normalized day 11 MN counts yielded 1.0%, equivalent to the basal level of MN observed at day 1 and 6 (Fig. 4b). We are confident our result is sufficiently accurate for data normalization using DMSO-treated tissues when determining the prevalence of MN in samples exposed to test articles using the optimized MN cytome assay protocol.

### Genetic toxicity assessment of 3D EpiIntestinal™ tissues exposed to reference genotoxin

**Fig. 5:**
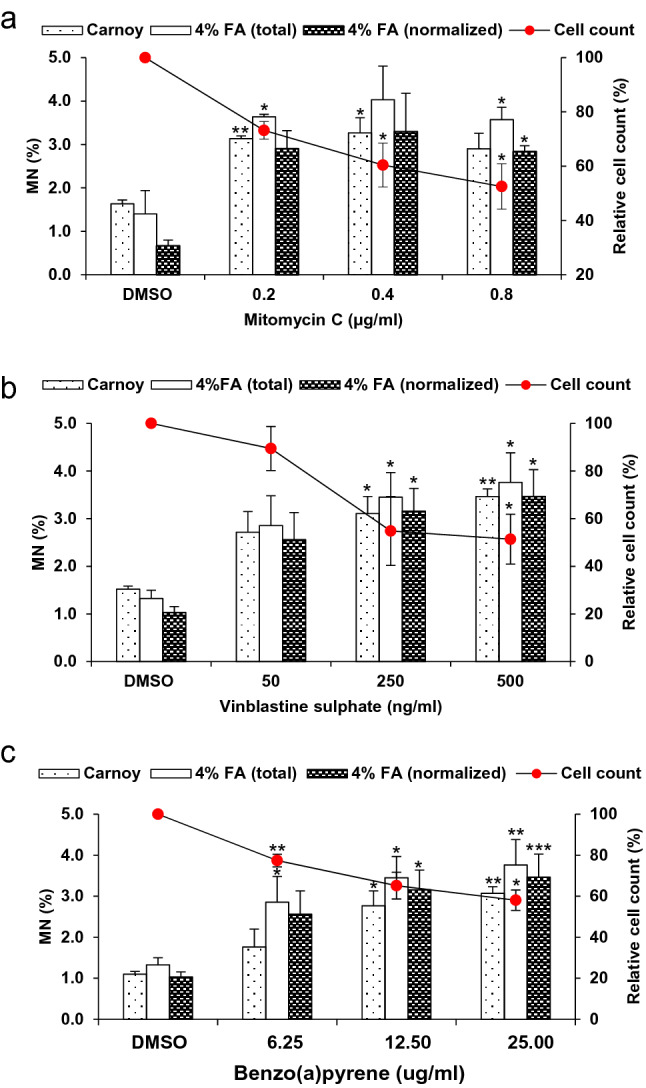
Genotoxicity assessment of reference chemicals exposed-3D EpiIntestinal™ tissue. Tissues were exposed to test articles: **a** mitomycin C, **b** vinblastine sulphate, and **c** benzo(a)pyrene; for 10 days via apical treatment. Genotoxicity was assessed until cell viability was decreased by approximately 50%. Tissues were dissociated to single cells, fixed in Carnoy’s fixative and 4% formaldehyde (FA) and scored according to the criteria described in “Materials and methods”. 4% FA (total) represents total MN scored from the fixed cells, while 4% FA (normalized) represents the population of MN after exclusion of DNA fragment(s) stemming from early apoptotic cells from the MN scoring. One thousand total cells were scored per microtissue to determine the frequency of MN induction. The results from three independent experiments are shown. Each experiment consisted of a single microtissue per test dose. *p < 0.05, **p < 0.01 and ***p < 0.001

To demonstrate the validity of the optimized MN cytome assay protocol to detect clastogenic and aneugenic compounds, and the effectiveness of the metabolic activation system, we exposed 3D EpiIntestinal™ tissues to reference genotoxins: clastogen (mitomycin c); clastogen requiring metabolic activation (benzo(a)pyrene); and aneugen (vinblastine sulphate). The responses of 3D EpiIntestinal™ tissue samples to the reference genotoxins is presented in Fig. 5. Significant decreases in cell population were observed in the 3D tissues exposed to doses ≥ 0.2 µg/ml mitomycin C, 500 ng/ml vinblastine sulphate and ≥ 6.25 µg/ml (benzo(a)pyrene). Significant dose-dependent induction of MN was also evident in 3D EpiIntestinal™ tissues exposed to any dose of these reference chemicals, compared to 3D EpiIntestinal™ tissues exposed to DMSO control samples. We assayed for active caspases-3 as a measure of basal MN, and to exclude DNA fragment(s) stemming from apoptotic cells from our MN analysis. We noted that consistency was high between measures of MN whether tissue samples were fixed in Carnoy’s fixative or 4% formaldehyde. In this study, EpiIntestinal™ tissues were dissociated and scored for nuclear anomalies (nuclear buds, binucleated, condensed chromatin, karyorrhectic, pyknotic and karyolytic cells), and for micronuclei in mononucleated cells (Table 1). We observed a dose-dependent increase in karyorrhectic cells in every tissue sample exposed to the genotoxins. Notably, the frequency of pyknotic and karyolytic cells was also increased in tissue samples exposed to vinblastine sulphate.

**Table 1:**
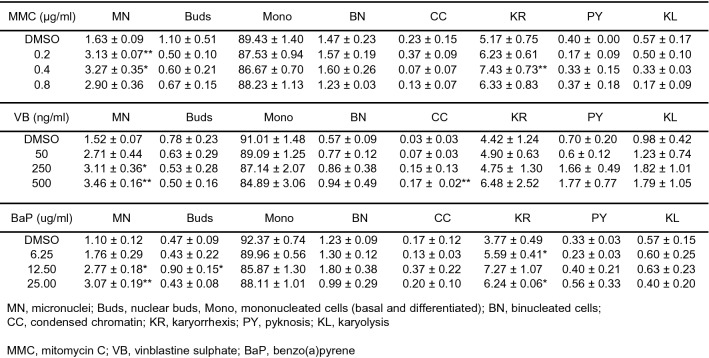
Cell phenotype and nuclear anomalies in 3D EpiIntestinal™ tissue

Reference genotoxins were applied to the apical surface of 3D EpiIntestinal™ tissues for 10 days, as described in “Materials and methods”. Tissues were dissociated into single cell suspensions and fixed in Carnoy’s fixative. One thousand total cells were scored per microtissue to determine the frequency of MN and cytome induction. The results from three independent experiments are shown. Each experiment consisted of a single microtissue per test dose. Data are reported as percentage (mean ± SE)

*MN* micronuclei, *Buds* nuclear buds, *Mono* mononucleated cells (basal and differentiated), *BN* binucleated cells, *CC* condensed chromatin, *KR* karyorrhexis, *PY* pyknosis, *KL* karyolysis, *MMC* mitomycin C, *VB* vinblastine sulphate, *BaP* benzo(a)pyrene

*p < 0.05 and **p < 0.01

### RICyt assay differentiates genotoxins from non-genotoxin

**Fig. 6:**
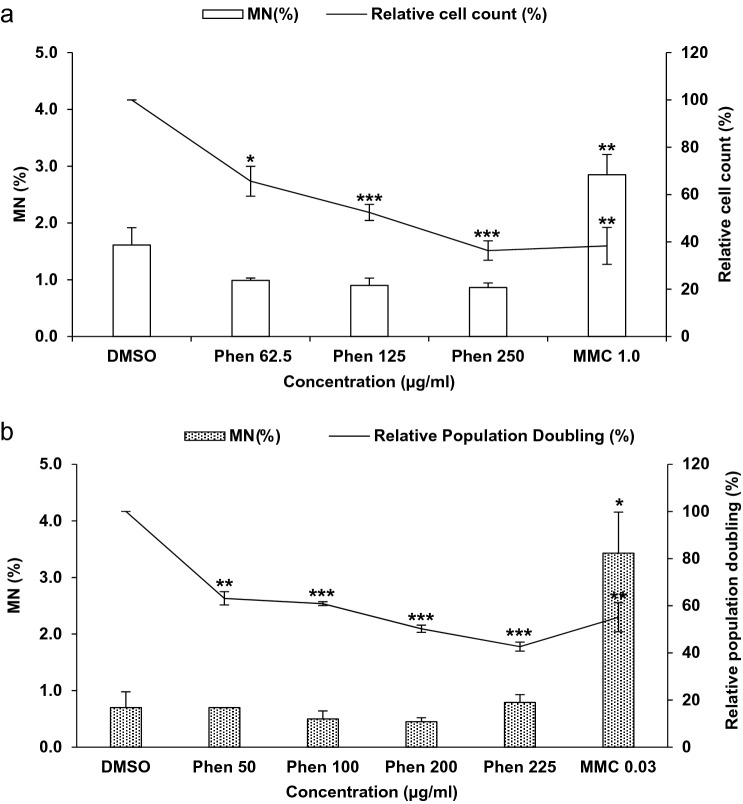
RICyt assay distinguishes genotoxins from non-genotoxin. **a** The genotoxin, mitomycin C, and non-genotoxic agent, phenformin HCl, were applied to the apical surfaces of 3D EpiIntestinal™ in vitro model tissues at various doses for 10 days. Tissues were dissociated into single cell suspensions, fixed in Carnoy’s fixative. One thousand total cells were scored per microtissue to determine the frequency of MN induction. Cell viability of treated 3D EpiIntestinal™ tissues are presented. The results from three independent experiments are shown. Each experiment consisted of a single microtissue per test dose. **b** A parallel study was conducted in which TK6 lymphocytic cells were exposed to mitomycin C and phenformin HCl at various doses for 24 h. Single cell suspensions were then fixed and scored. One thousand cells had been scored per treated sample for MN induction. Cell viability profile of treated TK6 cells are presented. The TK6 MN data were generated from independent duplicates. *p < 0.05, **p < 0.01 and ***p < 0.001

To further demonstrate the accuracy of RICyt assay in hazard identification, we exposed 3D EpiIntestinal™ tissues with mitomycin C, a known genotoxin, and phenformin HCl, a known non-genotoxin, using the regimen reported earlier (Fig. 2c). After 10 days’ exposure to mitomycin C, the prevalence of MN was found to increase by 3%, accompanied by a corresponding decline of 60% in the viable cell population (Fig. 6). In contrast, no genotoxicity was detected in tissues exposed to phenformin HCl. The prevalence of MN in phenformin HCl treated-tissue constructs was ~ 1%, indistinguishable from the DMSO control (1.5%). Although non-genotoxic, cells in 3D tissues exposed to phenformin HCl did exhibit dose-dependent cytotoxicity. Tissues exposed to 62.5, 125 and 250 µg/ml, exhibited 40%, 45% and 60% loss of viable cell populations, respectively (Fig. 6a). These data are comparable with observations acquired from a parallel study conducted using the 2D cell culture TK6 cell line. After 24 h-exposure to mitomycin C, the prevalence of MN in TK6 cells was assayed to be 3.4%, with a corresponding population decline of 55% (Fig. 6b). We did not detect any evidence of genotoxicity in TK6 cultures treated with phenformin HCl, and the prevalence of MN (~ 0.6%) were determined to be comparable across all concentrations tested, similar to cultures exposed to the DMSO control (0.7%). The cytotoxic profile was similar to 3D EpiIntestinal™, with 35%, 40%, 50% and 60% cytotoxicity in samples across the dose range tested. These results indicate that responses assayed in the 3D EpiIntestinal™ microtissue constructs are comparable with conventional 2D cell culture assays and that the RICyt assay is able to discriminate genotoxic reagents from non-genotoxic reagents.

## Discussion

The aim of this study was to evaluate whether the 3D EpiIntestinal™ platform of reconstructed microtissues is suitable for assessing the genotoxicity of ingested materials encountered by the human gut. We hypothesised that the human 3D EpiIntestinal™ tissue model is a suitable technology platform amenable to be adapted for evaluating the toxicity of ingested materials. To address this, we have developed a MN cytome assay protocol (RICyt assay). We report the findings of our evaluation, demonstrating that the 3D EpiIntestinal™ platform is capable of supporting hazard identification of orally ingested material. We show that 3D EpiIntestinal™ in vitro tissue is a micro-mimic of human small intestine: the live tissue model includes multiple cell types (enterocytes, paneth cells, M cells, tuft cells and intestinal stem cells), lamina propria, polarised phenotype, barrier functions and biochemical signature of native human intestinal tissue. The 3D EpiIntestinal™ model offers several advantages over current 2D monoculture protocols. It includes defined cell populations with polarised organisation characteristic of native intestine; the 3D EpiIntestinal™ model can be maintained in vitro for a reasonable period of time (> 10 days), remaining proliferative, differentiating and shedding from the apical surface for the duration of the assay. Constitutive intestinal cell shedding, a feature of the 3D EpiIntestinal™ model, makes the classical CBMN assay protocol unsuitable for this platform. Consequently, we developed and optimized the RICyt assay protocol as a comprehensive multiple endpoint-approach to measure DNA damage and cell death in 3D in vitro tissues. Having a low and reproducible baseline MN frequency, the 3D EpiIntestinal™ in vitro tissue model assures the RICyt assay is valid and accurate in detection of genotoxins and non-genotoxins, and producing more realistic and relevant responses to toxic agents than is available from protocols that utilise 2D monolayer cultures.

In vitro cells cultivated in 2D plastic vessels are neither physiological, nor systemic, and consequently fail to replicate the complexity inherent in intact tissues in vivo (Pridgeon et al. 2018). The evolution of in vitro 3D cell culture techniques and organotypic models is a promising approach to bridge the gap between 2D cell culture and in vivo organisms (OECD 2019, 2019). Recognising this, the OECD has published guidelines approving the use of reconstructed in vitro 3D tissue equivalents, including reconstructed human epidermis in vitro skin irritation test (OECD 439) (OECD 2019) and skin corrosion test (OECD 431) (OECD 2019). The 3D EpiIntestinal™ in vitro tissue used in this study offers relevant site of contact risk (apical surfaces of luminal villus), and accessibility to a comprehensive inventory of intestine-specific toxicity assays. Our data demonstrate that 3D EpiIntestinal™ maintains the complexity of native human intestinal epithelial architecture, its biochemistry, its physiology and its functionality, for up to 10 days in vitro. In addition, the 3D EpiIntestinal™ in vitro model has shown it is suitable for longer-term studies.

The MN assay was originally designed as a simple morphological assay to identify cells with post-mitotic chromosome breakage and/or whole chromosome loss, or dysfunctional chromosome segregation; events that requires mitosis, or meiotic division (Fenech 2007). Cyt-B is commonly used to block cytokinesis, leading to formation of BN cells, a convenient way to identify once-divided cells. However, we encountered a non-uniform block of cytokinesis because the 3D EpiIntestinal™ microtissues comprise unsynchronized cells in various stages of the cell cycle. While we could verify the 3D EpiIntestinal™ tissues maintained rapid cellular turnover of between 3 to 5 days (Umar 2010; Park et al. 2016; Creamer et al. 1961), extending treatment with Cyt-B up to 10 days did not improve the detection of post-mitotic cells. It is worth noting that intestinal epithelial homeostasis is maintained by a strict equilibrium between cell proliferation in the crypt, and cell shedding from the villus tip (Gregorieff et al. 2005; Byun et al. 2005). We interpret that the high cell proliferation evident in the 3D EpiIntestinal™ tissues coexists with a high rate of apical-luminal shedding, replicating the in vivo intrinsic mechanism maintaining intestinal barrier integrity and functionality (Günther et al. 2013; Doering et al. 2004; Gao and Wang 2009; Watson 2004). We suggest that active cell shedding likely resulted in the under-detection of post-mitotic cells; that the lower than expected prevalence of BN cells (~ 8.1%) we recorded in this study is not a true measure of endogenous cell proliferation in the 3D EpiIntestinal™ in vitro model.

In the adult gut, epithelial stem cells resident in the intestinal crypt, migrate upward, ascending adjacent villus as an epithelial ‘conveyor belt’. Proliferation occurring in the crypt is balanced by intimate interactions with the underlying matrix that orchestrates proliferation, migration, differentiation, and, ultimately, cell death (detachment-dependent apoptosis) when intestinal epithelial (i.e. EpiIntestinal™) cells are shed from intestinal villus into the lumen (Bullen et al. 2006; Williams et al. 2015). Unlike the GI tract, the 3D EpiIntestinal™ in vitro model lacks in vivo processes (peristalsis, fluid shear) that expel shed cells from the villus surface. This is a potential confounder of the model, as DNA fragments in apoptotic cells may be difficult to distinguish from MN (Baldassarre et al. 2000). Therefore, apoptosis is significant event with potential to affect measures of genotoxic potential of test chemicals. Others have applied differential labelling of chromatin of dead and dying cells with ethidium monoazide when scoring MN by flow cytometry (Avlasevich et al. 2006). Shed or shedding apoptotic intestinal cells have been identified using immunohistochemical techniques to enumerate early apoptotic cells expressing active caspase-3 (Bullen et al. 2006). In our model, we probed activated cleaved caspase-3 to detect early apoptotic cells prior to DNA fragmentation and shedding. Our data indicate the expression of active caspase-3 is persistent rather than transient; fluorescent signal intensity increased with DNA fragmentation, providing an early marker of apoptosis permitting us to distinguish DNA fragment(s) stemming from apoptotic cells from genuine MN. Our analyses of background MN compared to MN induced by reference genotoxins reveal that only a small fraction of DNA fragment(s) stemming from apoptotic cells were detected in 3D EpiIntestinal™ in vitro models. In our optimized protocols, frequent and thorough rinsing of the 3D tissues with HBSS effectively removed majority of basal apoptotic cells. We are confident that measures of MN in the 3D EpiIntestinal™ in vitro models following the RICyt assay protocols are not confounded by the presence of basal apoptosis.

In contrast to 3D tissue in vivo, the delivery of nutrients, growth factors and test reagents in vitro occurs via homogeneous culture media, such that all cells in 2D monolayer culture assays are exposed to identical conditions. The 2D cell culture method does not mimic the in vivo microenvironment of homeostatic tissue. Hence, cell populations cultivated in 2D tend to be synchronized, without a native extracellular matrix, and exist in a chronic state of proliferation (Tibbitt and Anseth 2009; Huh et al. 2011). In contrast, when cultivated in 3D culture systems, each cell interacts with a native-like pericellular tissue matrix, that serves to support cell differentiation and non-homogenous, stratified, 3D organisation. When grown in appropriate 3D organisation, cell morphology and physiology more closely recapitulate native tissue in vivo. It is, therefore, unsurprising that 3D cultures comprise unsynchronized cells in all stages of the cell cycle, including quiescence (G0), growth (G1/G2), proliferation (S), division (M), and also cell death (e.g. apoptosis, necrosis). Heterogeneity is characteristic of in vivo environments. We suggest that 3D tissue in vitro models represent a more authentic tissue technology platform for the assessment of genotoxicants.

Since we concluded that applying classical Cyt-B method to block cytokinesis was not viable in the 3D EpiIntestinal™ in vitro model, we adopted methodologies and concepts from the buccal micronucleus cytome assay, BMCyt. Originally described by Thomas et al. (2009), the BMCyt protocol assays the frequency of all cell types (binucleated cells, basal stem cells, atypical cells (condensed chromatin, karyorrhectic, pyknotic and karyolytic), rather than MN and nuclear buds, within in a minimum population of 1000 cells. The prevalence of MN and nuclear buds is scored from a minimum population of 2000 differentiated cells (Thomas et al. 2009). We modified this approach for our analysis of 3D EpiIntestinal™ model tissues, scoring basal, transitional and terminally differentiated cells as distinct populations; we subsequently pooled the three populations and reclassified them simply as ‘mononucleated cells’. For ease of scoring, MN and nuclear anomalies evident in each sample were scored at the same time we scored the three cell subpopulations. A minimum of 3000 cells were scored per test condition (i.e. the final MN data were tabulated from three independent experiments. Each experiment consisted of a single microtissue per test dose. A thousand cells were scored per microtissue.).

When challenged with benzo(a)pyrene without the use of exogenous metabolic activator (rat liver S9) the 3D EpiIntestinal™ in vitro model is at least partially metabolically competent (Ayehunie et al. 2018). We demonstrate that the modified BMCyt protocol, that we call RICyt assay, produces more realistic and relevant genotoxicity responses in 3D EpiIntestinal™ that are comparable with rodent gut micronucleus assay (Coffing et al. 2011) and reconstructed skin MN assay (Hu et al. 2009; Aardema et al. 2010) at similar doses. The RICyt assay demonstrated that the non-genotoxic cytotoxin, phenformin HCl, is inactive, whilst known reference genotoxicants are positive. These findings indicate that the RICyt assay protocol is a promising genotoxicity test platform.

At this stage, we have presented a proof-of-concept study of the RICyt assay as promising predictive tool in hazard identification of genotoxic materials. Although further work is needed to increase the validation of the assay, we show good correlation of our system with in vitro 3D and in vivo results (published by other groups) for the test materials. It is important to note, that 3D EpiIntestinal™ in vitro model is not fully identical to human native intestine: it lacks mucus secretion, commensal microbiota and peristalsis/fluid shear. Future work will investigate how to address these factors and assess their impact on the assay sensitivity and specificity. We are also currently validating the 3D EpiIntestinal™ in vitro model with a Comet assay protocol to detect additional genotoxicty endpoints to complement the RICyt assay protocol and enhance the utility of the model for genotoxicity testing. The MN-cytome scoring protocol described herein is a manual process and, as such, is time-consuming and requires trained and experienced users to assure the accuracy of the resulting data. Consequently, we have initiated collaborations to develop and validate automated scoring platforms to be used with the assay that we hope will aid progression towards high-content and/or high-throughput screening of multiple endpoints from samples subjected to the RICyt assay protocol. We believe our RICyt assay system offers a viable model bridging the gap between 2D cell and in vivo test systems and is well positioned to become a validated tool for assessing the safety of ingested materials, especially complex and novel food ingredients and technologies.

## Abbreviations

2D: Two dimensional
3D: Three dimensional
BMCyt: Buccal micronucleus cytome assay
BN: Binucleated
CBMN: Cytokinesis-block micronucleus assay
Cyt-B: Cytochalasin B
DAPI: 4′,6-Diamidino-2-phenylindole
DMEM: Dulbecco’s modification of minimal essential media
DMSO: Dimethyl sulphoxide
FITC: Fluorescein isothiocyanate
GI: Gastrointestinal
HBSS: Hanks balanced salt solution
HCl: Hydrogen chloride
IRB: Institutional Review Board
KCl: Potassium chloride
MN: Micronucleus
OECD: Organisation for Economic Co-operation and Development
PD: Population doubling
RHE: Reconstructed human epithelial
RICyt: Reconstructed Intestine Micronucleus Cytome
RPMI: Rosewell Park Memorial Institute
SE: Standard error
SEM: Scanning electron microscopy
TEER: TransEpithelial Electrical Resistance
